# Environmental Heterogeneity and Altitudinal Gradients Drive Darkling Beetle Diversity in an Alluvial Fan

**DOI:** 10.3390/insects16040388

**Published:** 2025-04-05

**Authors:** Min Zhao, Yuan Wang, Wenbin Yang, Yachao Zhu, Shuyu Zhang, Yongliang Liang, Guijun Yang

**Affiliations:** 1 School of Life Sciences, Ningxia University, Yinchuan 750021, China; 13409558544@163.com (M.Z.); oliver-wy@foxmail.com (Y.W.); nxd_ywb157@163.com (W.Y.); 17798441281@163.com (S.Z.); 2 Helan Mountain National Nature Reserve Administration of Ningxia, Yinchuan 750021, China; zyc451@foxmail.com (Y.Z.); liangyongliang@126.com (Y.L.); 3 Helan Mountain Forest Ecosystem Field Scientific Observatory of Ningxia Hui Autonomous Region, Yinchuan 750021, China

**Keywords:** alluvial fan, community diversity, Tenebrionidae, environmental factors, species diversity

## Abstract

Exploring the diversity and community structure of darkling beetles (Tenebrionidae) and the associated environmental factors on an alluvial fan provides useful insights into the ecology of these landscape features. This study investigated Chaqikou in the Helan Mountains, which features unique alluvial fan landforms. Sample plots (200 × 200 m) were established at three positions: the fan top, fan middle, and fan edge. From May to October 2023, pitfall traps were used to survey beetle community composition and its relationship with environmental factors. Significant variations were observed in species composition and diversity indices across different months and sample plots. Strongly xerophilous species exhibited broader ecological niche breadth, while moderately xerophilous species tended to distribute in the mid-to-upper segments of alluvial fans. Non-metric multidimensional scaling analysis revealed temporal shifts in community composition, with beta diversity analysis showing that species nestedness dominated from June to August, while species replacement was prominent in May, September, and October. Redundancy analysis indicated that environmental factors affecting species distribution varied by plot. On the landscape scale, altitude was the primary factor affecting beetle distribution. Variance partitioning analysis showed that topographic, soil, and vegetation factors explained 51.7%, 20.2%, and 9.4% of the variation in the beetle community, respectively. It is evident that altitudinal gradients shape ecological filtering pressures by creating multidimensional heterogeneity in topography, soil properties, and vegetation coverage. The adaptive matching between Tenebrionid species’ biological traits and environmental factors ultimately governs the spatial distribution patterns of darkling beetle diversity in alluvial fan desert grasslands.

## Simple Summary

Darkling beetles (Tenebrionidae), as typical indicator taxa in desert habitats, hold significant scientific value for understanding alluvial fan ecosystems. In 2023, we conducted a study at Chaqikou of Helan Mountain (renowned for its unique alluvial fan geomorphology) from May to October, establishing sampling plots at the fan top, fan middle, and fan edge to investigate Tenebrionid communities via pitfall trapping. Results indicated significant variations in species composition and diversity across months and sampling positions. Beta diversity analysis revealed temporal dynamics: community nestedness dominated from June to August, while species turnover prevailed in other months. At small scales, microhabitat heterogeneity profoundly influenced community diversity, whereas at landscape scales, elevation gradients shaped ecological filtering pressures through multidimensional heterogeneity in topography, soil properties, and vegetation coverage. The differential distribution patterns of darkling beetles across the alluvial fan emerged from species-specific biological traits and ecological adaptations to environmental factors.

## 1. Introduction

Darkling beetles exhibit a high level of species diversity, complex feeding behaviors, and wide geographical distribution, with approximately 60% of species found in desert or semi-desert regions [1]. As the indicator species of habitat degradation, darkling beetles play a crucial role in surface ecosystems and are significant for the restoration and rehabilitation of desert ecosystems [2,3]. Environmental heterogeneity influences the distribution, species diversity, structure, and functioning of ecosystems. Topography, as a key source of environmental spatiotemporal variability, regulates the spatial distribution of resources such as light, temperature, water, and soil nutrients through geomorphic processes [4]. Key topographic attributes, including altitude, slope, and aspect, significantly affect the habitat conditions for ground-dwelling beetles by influencing soil moisture levels [5]. On a small spatial scale, topographic factors play a critical role in shaping the distribution of ground-dwelling beetles in mountainous regions [6]. Darkling beetles are particularly abundant in areas with slopes less than 15° on sunny and semi-sunny slopes [7]. In arid desert habitats, darkling beetles exhibit a marked preference for microtopographic variations, particularly those associated with sand dunes, where shrub coverage and soil moisture differences emerge as key factors influencing beetle distribution [8]. Shrub coverage in desert areas provides essential shelter for darkling beetles, helping them avoid extreme temperatures, while also contributing to the creation of suitable ecological niches [9,10]. Additionally, plant roots serve as favorable breeding sites for ground-dwelling beetles, with larvae feeding on organic debris beneath the soil and the roots of living plants [11].

The dispersal ability of species is a key factor influencing community composition and distribution patterns. On a small spatial scale, environmental heterogeneity can constrain the spatial dispersal of beetles. Perennial vegetation belts act as barriers to short-distance movement in *Pterostichus melanarius* [12]. Tenebrionid beetles exhibit low-density distribution patterns in Poaceae-dominated vegetation zones at the 200 × 200 m spatial scale across arid-region alluvial fan habitats [13]. Increased habitat complexity reduces species richness of large-bodied Tenebrionid beetles [3]. The active dispersal ability of ground-dwelling beetles allows them to overcome environmental niche limitations and reach more suitable habitats, thereby reducing the influence of environmental heterogeneity on community dynamics [14]. The dispersal capacity of darkling beetles is linked to their functional traits [15]. Long-legged individuals of *Adesmia metallica* are primarily found in soil environments with low clay content and high rock and gravel content [16]. Short-winged carabid beetles have relatively weak flight abilities, whereas long-winged ground beetles are more capable of flight, leading to differences in *β* diversity across different habitats [17]. On semi-arid grasslands, the movement of darkling beetles is directional within a few meters, but over larger distances (hundreds of meters), their movement is typically constrained to the vicinity of their nests [18].

An alluvial fan is a type of piedmont geomorphic landscape commonly found in arid and semi-arid regions. Influenced by the mountain climate, large volumes of sediment carried by seasonal mountain runoff accumulate, thereby shaping the microtopography, soil, and vegetation on the surface of the alluvial fan [19]. The combined effects of flooding and deposition result in the formation of various microtopographic features, including high floodplains, groove beaches, alluvial mesas, and striated grooves, each exhibiting distinct soil characteristics [20]. The redistributive effect of topography on precipitation is particularly pronounced in alluvial fan environments, indirectly influencing vegetation distribution patterns [21]. On a larger scale, the distribution of *Ephedra przewalskii* populations on the alluvial fan at the southern foot of the Tianshan Mountains is closely linked to altitude, soil moisture, and the topographic wetness index. Furthermore, the driving factors for small-scale population aggregation differ across the fan’s top, middle, and bottom [22]. The coupling effects of soil compensatory erosion, particle transport, and deposition by seasonal rivers in arid desert alluvial fans significantly influence species diversity, plant growth, and spatial distribution [23,24,25]. The gravel cover on alluvial fans affects soil water content, surface runoff, erosion, nutrient loss, and vegetation cover, potentially altering the composition and spatial distribution of biological communities [26,27,28]. In semi-arid alluvial fan environments, a negative correlation has been observed between soil crust formation and gravel cover, with pebble debris being conducive to the development of soil crusts [29]. Despite this, relatively few studies have examined the relationship between the distribution of soil-dwelling animals and environmental factors in alluvial fan habitats. Soil sand content and vegetation structural complexity influence the distribution of ants, significantly enhancing the density of dominant species and affecting the overall community diversity [30]. Additionally, soil organic matter found in the roots of perennial and annual plants in alluvial fan habitats has been shown to increase nematode richness, whereas water distribution patterns appear to have no significant impact on nematode populations [31].

The Helan Mountains in northwest China serve as a critical geographical divide and ecological reserve. The eastern foothills of the Helan Mountains feature a relatively typical alluvial fan landscape, supporting rich biodiversity and serving as a vital water conservation area. In recent years, the rapid expansion of grape cultivation, the burgeoning ecotourism sector, and the frequent occurrence of seasonal mountain torrents at the eastern base of the Helan Mountains have posed significant challenges to the ecological balance of the alluvial fan landforms [32]. Darkling beetles are key species in the alluvial fan landforms, playing an essential role in surface processes, such as organic matter decomposition and energy flow [33], modification of soil physicochemical properties [34], and seed dispersal facilitation [35]. However, little attention has been paid to the community composition, diversity, and the interaction of darkling beetles with environmental factors in the alluvial fan landforms habitat. To address this gap, we examined the diversity and temporal dynamics of darkling beetle communities across different sites—fan top, fan middle, and fan edge—on the alluvial fan. Our analysis focuses on the underlying factors driving the diversity of darkling beetle communities at both small (site-specific) and large (entire fan) scales. In this study, our focal organism—the darkling beetles—serves as a dominant group in desert habitats. We investigated the spatiotemporal variations of Tenebrionidae community diversity across different sample sites on the alluvial fan from May to October and analyzed the underlying causes. Our aim is to elucidate the distribution patterns of Tenebrionidae diversity in this desert grassland ecosystem. Furthermore, this study provides a scientific foundation for seeking explanations to the maintenance mechanisms of Tenebrionidae community structure under the unstable conditions of alluvial fan ecosystems.

## 2. Materials and Methods

### 2.1. Study Area

The study area is situated in the Helan Mountains, located in the northwest of Ningxia, China (38°27′–39°30′ N, 105°41′–106°41′ E). This mountain range serves as a crucial geographical divide in China, with the Yinchuan Plain to the east and the Alxa Plateau to the west. The range extends approximately 200 km in length and 15–50 km in width, running from southwest to northeast. The average elevation of the Helan Mountains is around 2000 m, with its highest peak, Aobao Geda, reaching an altitude of 3556 m. The region experiences a temperate continental climate, with an average annual temperature of approximately −0.7 °C and annual precipitation ranging from 150 to 400 mm. Precipitation is relatively higher on the eastern slope of the mountains and predominantly occurs in the form of intense rainstorms. Vegetation is diverse, displaying a distinct vertical zonation across the mountain range [36]. At the eastern foot of the Helan Mountains, a typical alluvial fan landform is present, with dominant soil types, including calcareous gravel and aeolian sandy soils. The vegetation is primarily desert grassland, characterized by drought-tolerant herbaceous species such as *Stipa capillata* and *Neotrinia splendens*, along with sparse occurrences of small shrubs [19].

### 2.2. Sample Sites and Selection

**Figure 1:**
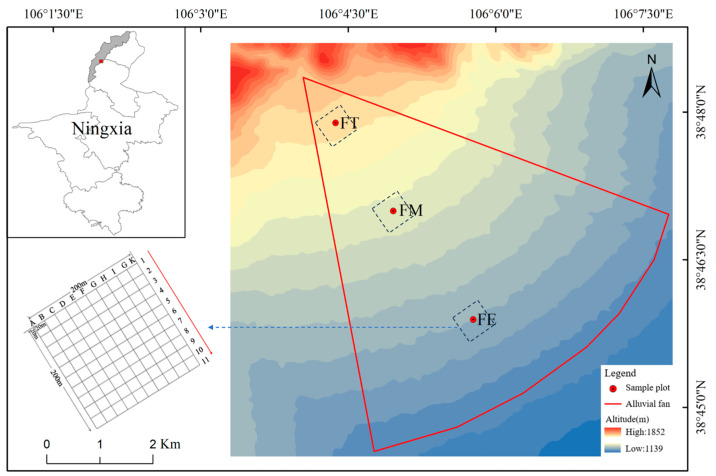
Location and setup of research sample plots in the desert grasslands of the Chaqikou alluvial fan.

The study was conducted in the desert grasslands of the Chaqikou alluvial fan, located at the eastern foot of the Helan Mountains (38°48′20″ N, 106°04′03″ E). The elevation at the mouth of the gully is 1550 m. The alluvial fan extends approximately 10 km in length, and the elevation varies by about 300 m from the fan top to the fan edge. To capture the variation along the elevation gradient, three research sample plots were established: FT (at the fan top), FM (at the fan middle), and FE (at the fan edge), as depicted in Figure 1. We used the handheld GPS (Zhuolin Electronic Technology Co., Ltd., Wanguo Building, Yaohai District, Hefei, China, model: A6) to ensure that the elevation difference between adjacent sample plots is approximately 100 m, and the horizontal distance is greater than 2 km. Each sample plot, covering an area of 4 ha (200 m × 200 m), was divided into 100 cells of 20 m × 20 m, resulting in a total of 121 grid intersection points across all plots. The variations in temperature and precipitation at the sampling site are presented in Table 1.

### 2.3. Collection of Darkling Beetles

Darkling beetles were sampled using the pitfall trapping method improved by Guijun Yang [13]. A single trap was installed at each grid intersection within the sampling plot. The attractant used in the traps was a concoction of vinegar, sugar, alcohol, and water in a mass ratio of 2:1:1:2, with an approximate volume of 500 mL. Each sampling plot contained 121 sampling points, resulting in a total of 363 traps being deployed. A total of six samplings were carried out in this study, with each sampling conducted monthly from May to October 2023. During each sampling period, the traps were left for seven days. Subsequently, the adult beetles were collected and preserved in 75% ethanol. The collected beetles were identified based on relevant literature [1,37,38], with reference to the insect specimens stored in the Entomological Specimen Room of the Helan Mountain Nature Reserve.

### 2.4. Investigation of Environmental Factors

Topographic factors included six variables: elevation (E), plan curvature (PLC), topographic wetness index (TWI), profile curvature (PRC), slope (S), and slope aspect (SA). These variables were extracted from the regional Digital Elevation Model (DEM) using ArcGIS 10.8.

Vegetation factors were represented by five variables. Specifically, three plant quadrats were established at each beetle sampling point: two quadrats measuring 5 m × 5 m (for shrubs) and one quadrat measuring 1 m × 1 m (for herbs). Within these quadrats, the following variables were measured: plant species richness (PSR), plant diversity (PD), vegetation height (VH), vegetation coverage (VC), and plant biomass (PB).

Soil factors included 12 variables related to surface gravel cover and the physical and chemical properties of the soil. At each sampling point, three soil samples were randomly collected from a depth of 15 cm beneath the surface within the vegetation investigation quadrat. These samples were then mixed to measure the following variables: total nitrogen (TN), total phosphorus (TP), soil organic matter (SOM), pH, soil water content (SWC), soil electrical conductivity (EC), and soil bulk density (SBD) [39].

Additionally, a 1 m × 1 m gravel quadrat was established within 50 cm of each beetle sampling point. Vertical photographs (with a resolution of more than 2000 pixels) of the quadrat were captured and processed using Photoshop 2021. From these images, Feret’s diameter (DF), aspect ratio (AR), and circularity degree (CD) of gravel particles larger than 1 cm were calculated using Digimizer 5.7. The gravel density (GD) and gravel coverage (GC) within the quadrat were quantified [40].

### 2.5. Data Analysis

#### 2.5.1. Analysis of the Diversity of Darkling Beetle Communities

The dominance of the species was classified based on the number of individuals within the darkling beetle community in the sample plots. Species whose abundance accounted for less than 1% of the total number of individuals in a plot were considered rare species; those with an abundance between 1% (inclusive) and 5% were classified as common species; species with abundances ranging from 5% (inclusive) to 10% were classified as subdominant species; and species comprising 10% (inclusive) or more of the total individuals were considered dominant species [41]. The Levins’ niche breadth index was employed to characterize the niche occupation patterns of darkling beetle species across alluvial fan scales [42]. Quantification was implemented through the ‘spaa’ package (version 0.2.5) within R 4.3.3.

In this study, we applied the Margalef richness index, Shannon–Wiener diversity index, Pielou evenness index, and Berger–Parker dominance index. These indices comprehensively assess the composition of darkling beetle communities across multiple dimensions, including species richness, evenness, etc. We attempted to analyze the structural changes of the darkling beetle community more accurately under different environmental conditions, thereby revealing the relationship between environmental factors and community characteristics [43].

#### 2.5.2. Non-Metric Multidimensional Scaling Analysis

The species composition of darkling beetles at the sampling points across different months was used as an attribute for a non-metric multidimensional scaling (NMDS) analysis. The ordination diagram was generated using Canoco 5.12 to examine and compare the temporal relationships within the darkling beetle communities.

#### 2.5.3. Beta Diversity Analysis

The beta diversity of the darkling beetle community was decomposed into turnover and nestedness components using the Sorensen dissimilarity index [44]. Beta diversity decomposition was conducted using the beta.div.comp function from the ‘adespatial’ package in R version 4.3.2.

#### 2.5.4. Analysis of the Relationship Between the Diversity of Darkling Beetle Communities and Environmental Factors

The Mantel test was applied to assess the correlation between darkling beetle species and environmental factors, with the analysis and visual representation conducted using the ‘vegan’ package in R 4.3.2. Redundancy analysis (RDA) was performed to examine the relationship between species richness and environmental variables, while variance partitioning analysis (VPA) was used to determine the independent and combined explanatory contributions of environmental factors to beetle species richness. Both RDA and VPA were carried out using Canoco 5.12. The forward selection procedure was employed to evaluate the contribution and significance of various environmental factors to beetle distribution. Prior to analysis, species abundance data and environmental factor data were transformed using the lg(x + 1) function.

## 3. Results

### 3.1. Composition of Darkling Beetle Communities

A total of 2567 darkling beetles, belonging to 9 genera and 12 species, were collected (Table 2). Statistics were carried out on the number of darkling beetles collected in the three sample plots. The dominant species were *Microdera kraatzi* (36.66%), *Anatolica planata* (16.09%), and *Anatolica pandaroides* (13.71%), which together accounted for 66.46% of the total individuals. Subdominant species included *Scytosoma pygmaeum*, *Scytosoma dissilimarginis*, *Anatolica polita*, *Blaps femoralis*, and *Scleropatrum horridum*. *Penthicus alashanicus* and *Opatyum subaratum* were classified as common species, while *Melanesthes rugipennis* and *Crypticus rufipes* were considered rare species.

Notable differences in dominant species were observed among the sample plots. In the plot FT, *M. kraatzi* and *S. horridum* were the dominant species. In the plot, FM, *M. kraatzi*, *A. planata*, *A. pandaroides*, and *B. femoralis* emerged as dominant species. In the plot FE, *A. planata*, *A. pandaroides*, and *M. kraatzi* were the dominant species.

### 3.2. Diversity of Darkling Beetle Communities

#### 3.2.1. Temporal Dynamics of the Diversity of Darkling Beetle Communities in Different Sample Plots

**Figure 2:**
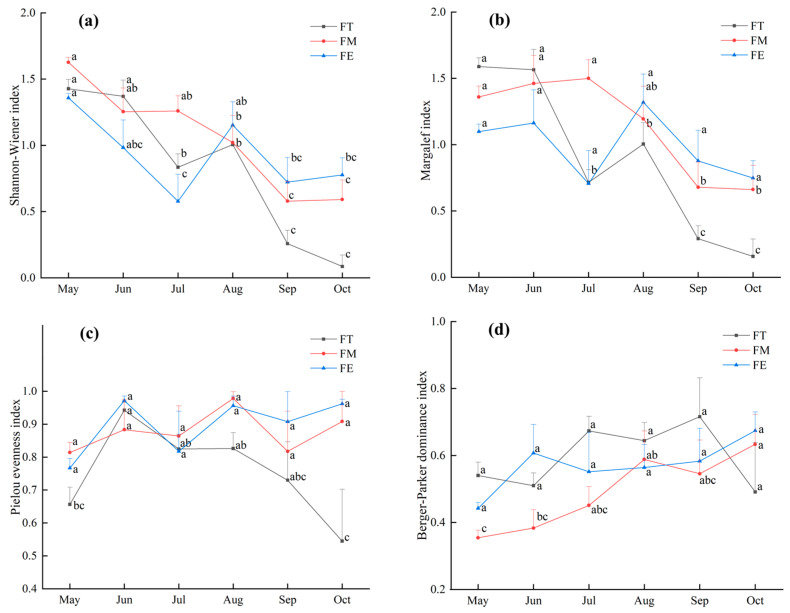
Temporal changes of darkling beetles’ diversity indices in different sample plots: (**a**) Shannon–Wiener diversity index, (**b**) Margalef richness index, (**c**) Pielou evenness index, and (**d**) Berger–Parker dominance index. Different lowercase letters represent significant difference at 0.05 level.

The Shannon–Wiener diversity index and Margalef richness index for the darkling beetle communities in the different sample plots generally exhibited a declining trend over time (Figure 2a, b). However, both indices showed an upward trend in sample plots FT and FE from July to August. The Pielou evenness index fluctuated significantly throughout the study period, with the index in sample plot FT remaining relatively low overall (Figure 2c). In contrast, the Berger–Parker dominance index demonstrated a steady upward trend over time (Figure 2d). Notably, the Berger–Parker dominance index in sample plot FM remained relatively low.

**Figure 3:**
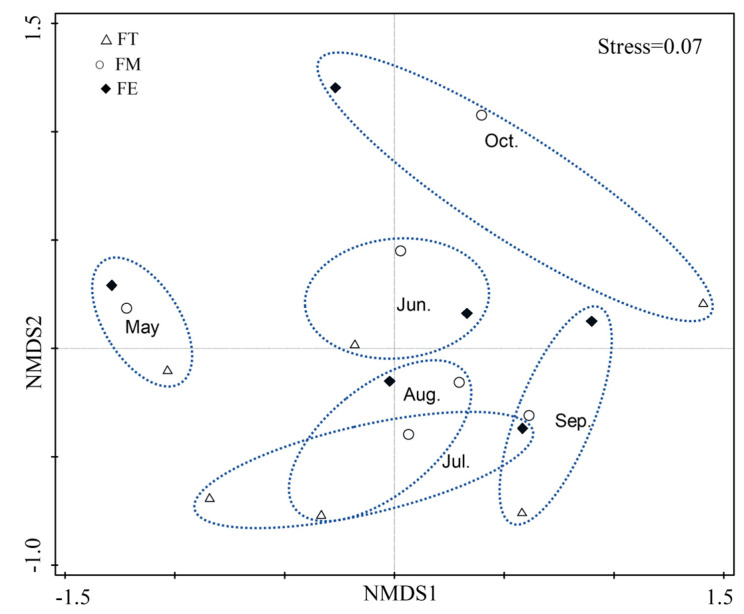
Non-metric multidimensional scaling analysis (NMDS) of darkling beetle communities in different months. The dashed lines represent the confidence ellipses between different months for the three sample plots. Hollow triangles denote the fan top sample plot, hollow circles represent the fan middle, and black squares indicate the fan edge.

The NMDS analysis revealed a stress value of 0.07 for the darkling beetle communities across different months, which indicated meaningful explanatory power (Figure 3). Significant differences were observed between the darkling beetle communities in May, June, and October and those in the other months. The confidence intervals for July, August, and September overlapped, suggesting that the community structures of darkling beetles in these three months were relatively similar.

#### 3.2.2. Beta Diversity and Its Components of Darkling Beetle Communities

**Figure 4:**
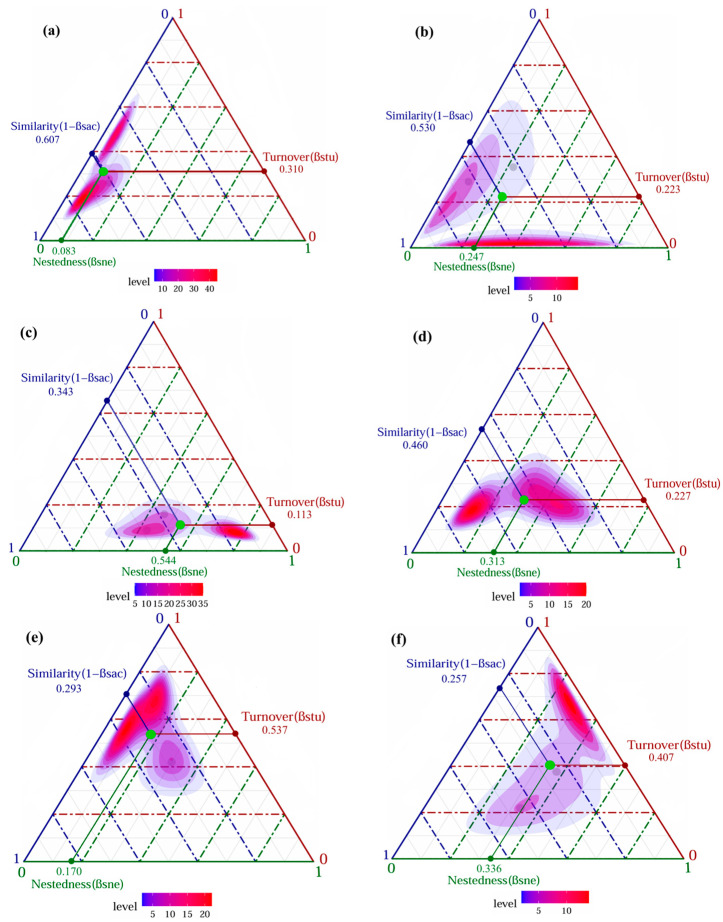
The ternary plot illustrates the beta diversity and its two components across different months. The green dots indicate the mean values for each month. Panels (**a**–**f**) correspond to the relationships between the components at the sampling times in May, June, July, August, September, and October, respectively. The purple dots represent different sample plots.

In general, the beta diversity for darkling beetle communities exhibited gradual increase over the months (Figure 4). Notably, the turnover components in June, July, and August were smaller than the nestedness components, indicating that nestedness played a dominant role in the beta diversity of darkling beetles during this period. Conversely, in May, September, and October, the turnover components outweighed the nestedness components. Furthermore, significant seasonal variation was observed in the contribution rates of turnover components to the beta diversity, with distinct differences between the summer months (June, July, and August) and the remaining months (May, September, and October).

### 3.3. The Relationship Between the Distribution of Darkling Beetle Species and Environmental Factors

#### 3.3.1. The Correlation Between Species Abundance and Environmental Factors

**Figure 5:**
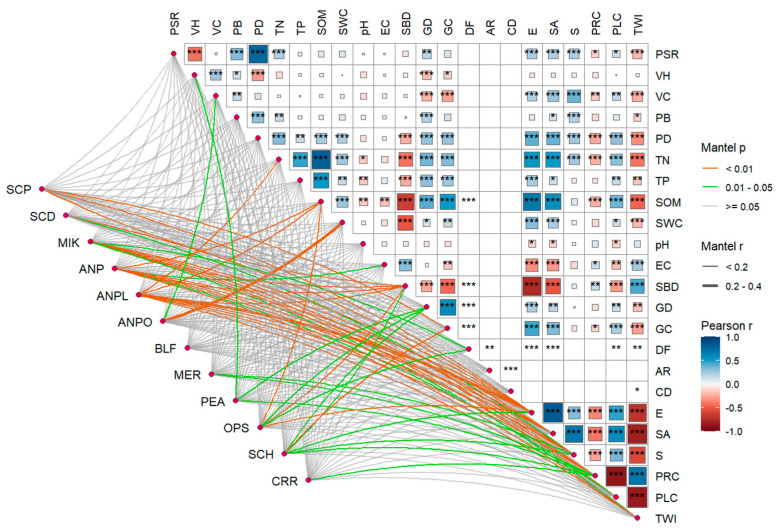
Mantel test of the relationship between darkling beetle species and environmental factors. The size of the blocks represents the Pearson correlation coefficients between different environmental factors. Larger and darker-colored blocks indicate the higher value of Pearson ∣r∣. The abbreviations for species are as follows: SCP: *Scytosoma pygmaeum*, SCD: *Scytosoma dissilimarginis*, MIK: *Microdera kraatzi*, ANP: *Anatolica pandaroides*, ANPL: *Anatolica planata*, ANPO: *Anatolica polita*, BLF: *Blaps femoralis*, MER: *Melanesthes rugipennis*, PEA: *Penthicus alashanicus*, OPS: *Opatyum subaratum*, SCH: *Scleropatrum horridum*, and CRR: *Crypticus rufipes*. PSR: plant species richness, VH: vegetation height, VC: vegetation coverage, PB: plant biomass, PD: plant diversity, TN: total nitrogen, TP: total phosphorus, SOM: soil organic matter, SWC: soil water content, EC: soil electrical conductivity, SBD: soil bulk density, GD: gravel density, GC: gravel coverage, DF: Feret’s diameter, AR: aspect ratio, CD: circularity degree, E: elevation, SA: slope aspect, S: slope, PRC: profile curvature, PLC: plan curvature, and TWI: topographic wetness index. The number of asterisks indicates different levels of significance: * indicates significance at the 0.05 significance level, ** indicates significance at the 0.01 significance level, and *** indicates significance at the 0.001 significance level.

The Mantel test analysis revealed that the abundance of darkling beetle species was more strongly associated with topographic and soil factors (Figure 5). The dominant species *M. kraatzi* exhibited significant correlations with elevation (E), slope aspect (SA), slope (S), profile curvature (PRC), plan curvature (PLC), and soil bulk density (SBD) (*p* < 0.01). *A. pandaroides* showed significant correlations with E, SA, S, PRC, SBD, topographic wetness index (TWI), and soil organic matter (SOM) (*p* < 0.01). The distribution of *A. planata* was significantly correlated with E, SA, S, PRC, SBD, total nitrogen (TN), SOM, and TWI (*p* < 0.01). Gravel cover (GC) had a significant impact on the distribution of *S. horridum* (*p* < 0.01).

#### 3.3.2. Influencing Factors of the Distribution of Darkling Beetle Species in Different Sample Plots

**Figure 6:**
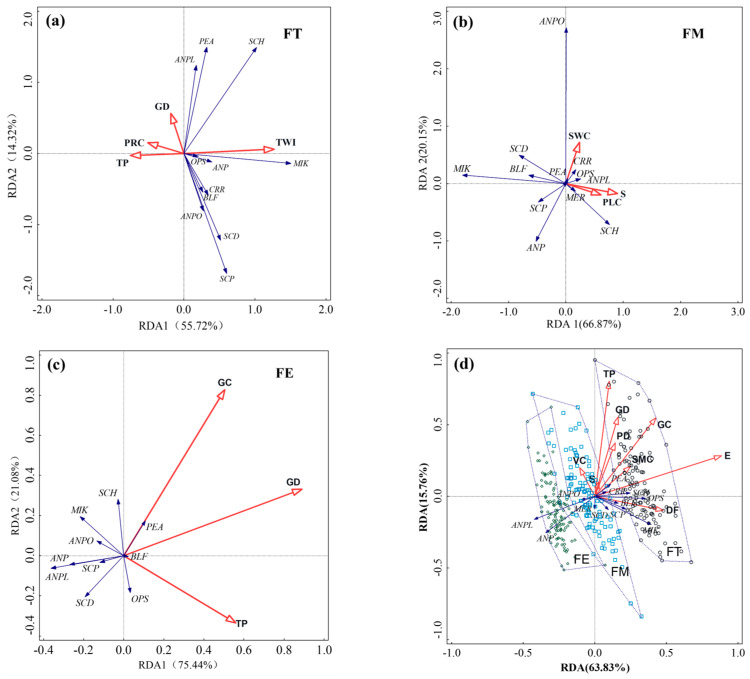
RDA of the distribution of darkling beetles and environmental factors (**a**) in sample plot FT; (**b**) in sample plot FM; (**c**) in sample plot FE; and (**d**) in the entire fan scale of alluvial fan. The red lines with arrows represent the corresponding environmental factors, and the blue lines represent different darkling beetle (Tenebrionidae) species. In panel (**d**), the dashed polygons represent different sample plots, with the different points indicating the pitfall traps from each corresponding sample plot.

Based on the small-scale spatial analysis within a 200 m × 200 m area, redundancy analysis (RDA) was performed to examine the distribution of darkling beetles and environmental factors in the three sample plots. In the sample plot FT, four factors were found to significantly influence beetle distribution (*p* < 0.05). The topographic wetness index (TWI) emerged as the primary factor, contributing 31.0% (*F* = 9.9, *p* = 0.002), followed by profile curvature (PRC) (10.1%), total phosphorus (TP) (7.1%), and gravel density (GD) (6.8%). The distribution of the dominant species, *M. kraatzi*, was positively correlated with TWI but negatively correlated with the other three factors. The distribution of *S. horridum* was positively correlated with TWI and GD, while being negatively correlated with PRC and TP (Figure 6a). In sample plot FM, three factors significantly influenced beetle distribution (*p* < 0.05): slope (S) (contributing 14.7%, *F* = 3.1, *p* = 0.012), plan curvature (PLC) (13.1%, *F* = 2.8, *p* = 0.022), and soil water content (SWC) (10.6%, *F* = 2.3, *p* = 0.032). The dominant species, *M. kraatzi* and *B. femoralis,* were negatively correlated with all three factors, while *A. planata* was positively correlated with all three. *A. pandaroides* showed a positive correlation with SWC and a negative correlation with S and PLC (Figure 6b). In the sample plot FE, three factors were significantly correlated with beetle distribution (*p* < 0.05): GD (23.0%, *F* = 6.8, *p* = 0.002), TP (7.2%, *F* = 2.1, *p* = 0.04), and gravel cover (GC) (6.7%, *F* = 2.0, *p* = 0.05). The dominant species *A. planata*, *A. pandaroides*, and *M. kraatzi* were all negatively correlated with these three factors (Figure 6c).

#### 3.3.3. Influencing Factors of the Distribution of Darkling Beetle Species in Fan Landscapes

The RDA of beetles and environmental factors across the entire fan landscape revealed that the cumulative contribution rate of the X and Y axes was 79.59%, with the first axis accounting for 63.83% (*F* = 2.0, *p* = 0.002) (Figure 6d). Among the nine factors significantly influencing the distribution of darkling beetle species (*p* < 0.05), vegetation coverage (VC) was positioned on the negative side of the RDA1 axis, while the remaining factors were situated on the positive side. Elevation (E) was the primary factor affecting species distribution, contributing 42.3% (*F* = 32.8, *p* = 0.002). Among soil factors, total phosphorus (TP) contributed the highest (8.98%), while VC (4.29%) and plant diversity (PD) (4.28%) were the vegetation factors with the highest contribution rates. The darkling beetle communities in sample plots FT, FM, and FE were arranged along the first axis from positive to negative, with partial overlap between FM and FE, suggesting relatively high community similarity. The dominant species, *M. kraatzi,* was positively correlated with E and negatively correlated with TP, VC, and PD. *A. planata* showed a negative correlation with E, VC, and TP, while *A. pandaroides* was negatively correlated with E, TP, VC, and PD. The distribution of *M. kraatzi* was primarily associated with sample plot FT, while *A. planata* and *A. pandaroides* were more associated with sample plots FM and FE.

**Figure 7:**
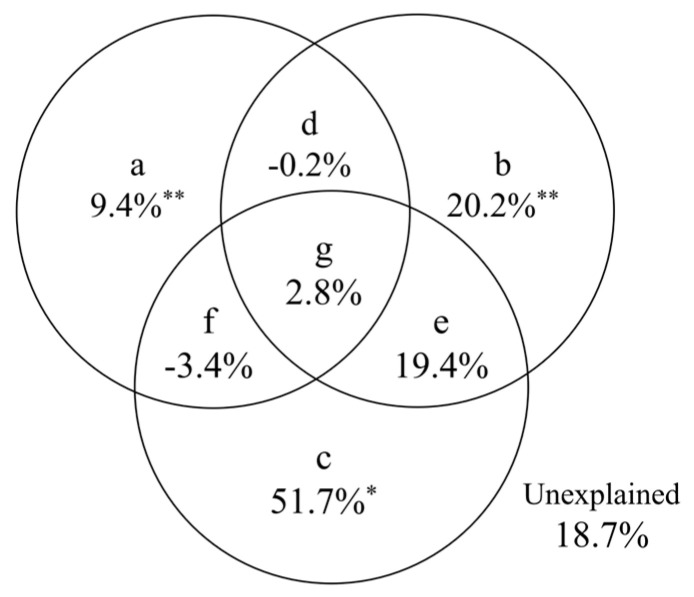
The variance partitioning analysis (VPA) of environmental factors on the variation of darkling beetle communities. The lowercase letters represent the following meanings: (**a**) represents the vegetation factor, (**b**) represents the soil factor, and (**c**) represents the topographic factor, (**d**–**f**) indicate the shared explanatory rates between two factors, and (**g**) represents the combined explanatory rate of all three factors. (* *p* < 0.05, ** *p* < 0.01).

The VPA revealed that vegetation factors (a), soil factors (b), and topographic factors (c) explained 9.4%, 20.2%, and 51.7% of the variation in the darkling beetle community, respectively. These three factors accounted for 81.3% of the total variation in the community, with 18.7% remaining unexplained (Figure 7).

## 4. Discussion

### 4.1. Dominant Species of Darkling Beetle Communities in Alluvial Fan Landforms

In this study, the Pimeliinae insects investigated belong to strongly xerophilous species, while Tenebrioninae and Diaperinae insects are moderately xerophilous species [1,37,38]. Insects of the Pimeliinae are keystone hosts in desert habitats and serve as the dominant insect group in arid environments [1,45,46], exhibiting a broad niche breadth within the alluvial fan landscape of this study. The findings of this study indicate that the dominant species within the darkling beetle community across the alluvial fan landscape were *M. kraatzi*, *A. planata*, and *A. pandaroides*, all of which belong to the subfamily Pimeliinae. The observed variations in dominant species across different plots reflect the microhabitat heterogeneity from the top to the edge of the alluvial fan. We found that *M. kraatzi* preferred to aggregate under slates in a radial arrangement with their heads pointing outward, which is consistent with the records by Guodong Ren et al. [37]. Therefore, *M. kraatzi* showed higher abundance in the gravel-covered plot FT. In contrast, *A. planata* and *A. pandaroides* were dominant in the FM and FE plots, indicating their preference for the middle and lower sections of the alluvial fan. Species such as *S. horridum* and *B. femoralis*, belonging to the subfamily Tenebrioninae, were dominant in the FT and FM plots, respectively. Tenebrioninae beetles are generally adapted to arid environments with non-sandy soils, highlighting their ecological niche preferences. In the FE plot, all dominant species were members of the Pimeliinae subfamily, likely due to the favorable sandy soil conditions at the fan edge, which provided a more suitable habitat for these species.

### 4.2. Diversity Changes of Darkling Beetle Communities in Alluvial Fan Landforms

The NMDS analysis of darkling beetle communities across different months revealed distinct seasonal patterns, with May and October representing spring and autumn community compositions, respectively. Seasonal precipitation likely played a significant role in shaping the habitats of soil-dwelling organisms within the alluvial fan. Rainfall in the study area primarily occurred in mid-to-late August, with additional precipitation in September. The community compositions of darkling beetles in September and from June to August were relatively similar, reflecting the characteristics of summer assemblages. These findings underscored the clear temporal patterns within the summer season of darkling beetle communities, demonstrating the resilience and stability of these communities in response to environmental fluctuations.

The decomposition of beta diversity provides valuable insights into species turnover and nestedness [44], reflecting changes in community composition and diversity across time and space [47]. During spring (May) and autumn (September and October), species turnover was the dominant component of beta diversity, indicating significant species replacement during these periods. This may be attributed to the strong dispersal capabilities of the dominant species, particularly those belonging to the subfamily Tentyriini (e.g., *M. kraatzi*, *A. pandaroides*, *A. planata*, *A. polita*), which possess elongated abdomens and legs that enhance their mobility [15,37]. These traits likely facilitated their movement across different microhabitats within the alluvial fan, enabling them to adapt to changing environmental conditions.

In contrast, during the summer months (June, July, and August), nestedness was the dominant component of beta diversity, suggesting a mosaic pattern of species assemblages. This pattern is unlikely to be due to a reduction in species richness but may instead reflect the life history traits of darkling beetles. Many adult beetles die successively after laying eggs in spring, and their larval development stage is prolonged [1,37,38]. Since this study focused on adult beetles, the phenomenon that some species had a small number or were even apparently absent in some plots after summer might have influenced the observed community composition.

### 4.3. Analysis of the Causes for the Distribution of Darkling Beetle Species in Alluvial Fan Landforms

The RDA analysis revealed that the environmental factors influencing darkling beetle distribution varied across sample plots. In the FT plot, TWI, PRC, TP, and GD were significant factors. In the FM plot, S, PLC, and SWC played key roles. In the FE plot, GD, TP, and GC were the primary drivers. These findings highlight the microhabitat heterogeneity along the altitudinal gradient from the fan top to the fan edge. Notably, vegetation characteristics did not significantly influence species distribution in the desert grassland microhabitats of this study.

Based on the RDA analysis of darkling beetle communities’ distribution across the alluvial fan landscape, the communities from the three sample plots exhibited distinct arrangements along the ordination axis (x-axis), reflecting the gradient differences in nine environmental factors that significantly influenced darkling beetles’ distribution. Elevation emerged as the primary factor, governing habitat heterogeneity across the plots. Communities in plots FM and FE showed greater similarity, whereas those in plot FT (located at the fan’s upper reaches) displayed substantial divergence. This spatial variation may stem from species-specific responses to environmental conditions: species such as *M. kraatzi*, *S. horridum*, *P. alashanicus*, and *O. subaratum* abundant at higher elevations with greater gravel coverage and soil moisture, favoring microhabitats beneath rocks where gravel mulch provided shaded, humid conditions. In contrast, *A. pandaroides* and *A. planata* were more abundant in mid-to-lower fan areas with lower elevation, reduced gravel cover, and drier soils, suggesting their superior arid adaptability. Species dispersal capacity also shaped community patterns. While darkling beetles in this study exhibited relatively weak dispersal capabilities both within the soil matrix and across the surface substrate, Pimeliinae (Tentyriini) demonstrated faster movement and larger activity ranges compared to Tenebrioninae and Diaperinae [1,37], likely linked to functional traits and ecological adaptations requiring further investigation. At the landscape scale of this study, no significant dispersal barriers existed for darkling beetles. Environmental heterogeneity and species-specific ecological adaptations drove immigration-emigration dynamics, ultimately shaping community composition and spatial distribution patterns [12,13,48].

The VPA conducted at the alluvial fan scale revealed that topographic, soil, and vegetation factors explained 51.7%, 20.2%, and 9.4% of the variation in darkling beetle communities, respectively. Altitude emerged as the dominant factor driving habitat variation, leading to differences in topography and vegetation across the altitudinal gradient [23]. Seasonal precipitation and surface runoff, characteristics of alluvial fans, significantly influenced microtopography, gravel coverage, and soil structure, which in turn affected vegetation types and density [49]. Variations in soil nutrients were also observed across microtopographic features such as high floodplains, groove beaches, and alluvial mesas [20].

Among soil factors, TP was the most significant driver of darkling beetle distribution. Further investigation is needed to better understand the distribution patterns of darkling beetles in different microtopographies. Gravel coverage and gravel density also played roles, albeit with lower contribution rates. Surface gravel positively influenced soil water infiltration and reduced evaporation [26,28], thereby improving soil structure and providing suitable habitats for ground-dwelling beetles. Among vegetation-related factors, VC and PD exhibited the highest contribution rates. The “fertility island effect” of vegetation [50] and the “canopy effect” [10,11] likely created nutrient-rich and safe soil environments for beetles in desert habitats.

The VPA indicated that topographic, soil, and vegetation factors collectively explained 81.3% of the variation in darkling beetle distribution, leaving 18.7% unexplained. Soberón [51] proposed a framework for understanding species diversity and distribution at multiple spatial scales, emphasizing the interplay of environmental heterogeneity, biological dispersal, and species interactions. On smaller spatial scales, biological interactions, such as interspecific competition among darkling beetles [13,52,53] and predation pressure from natural enemies [54,55], may play significant roles in shaping community dynamics. These factors warrant further investigation to fully elucidate the mechanisms underlying darkling beetle distribution in alluvial fan landscapes.

## 5. Conclusions

This study provided a comprehensive assessment of the community composition of darkling beetles in the alluvial fan landforms. The results revealed that the community similarity between the fan middle and the fan edge was the highest, though the dominant species at the fan top, fan middle, and fan edge were not entirely consistent. The NMDS analysis of darkling beetle communities across different months highlighted distinct seasonal patterns in community composition. Beta diversity analysis indicated that from June to August, species nestedness predominated in shaping the community structure, whereas species replacement played a more significant role in May, September, and October.

At the small-scale landscape level, the microhabitats influencing the distribution of darkling beetle communities in the fan top, fan middle, and fan edge of alluvial fan desert grasslands exhibit distinct differences. This heterogeneity is primarily reflected in the spatial gradient differentiation of microtopography, gravel coverage, and soil texture. At the alluvial fan landscape scale, elevation emerges as the core environmental factor driving the distribution pattern of darkling beetles, shaping gradient heterogeneity in topography, soil, and vegetation to form multidimensional ecological filtering pressures. The biological traits of different darkling beetle species form significant ecological adaptation associations with environmental heterogeneity, thereby leading to differentiated distribution patterns at the alluvial fan landscape scale. Notably, in desert grasslands with limited food resources, interspecific competition and predation pressure from natural enemies may also be factors that need to be considered.

## Figures and Tables

**Table 1:** Monthly variation of temperature and precipitation at the sample plots.

Month	Precipitation (mm)	Maximum Temperature (°C)	Mean Temperature (°C)	Minimum Temperature (°C)
April	9	17.3	9.8	2.3
May	18	23.6	16.4	9.1
June	42	27.5	20.8	14.1
July	67	29.1	22.8	16.5
August	51	27.2	21.1	15.1
September	25	22.5	15.9	9.3
October	13	16.0	9.0	2.0

**Table 2:** Species composition and individual proportion of darkling beetle communities sampled from May to October in the Chaqikou alluvial fan.

Species		Abbreviation	FT		FM		FE		Percentage (%)	Levins’ Index
			Frequency *	Proportion	Frequency	Proportion	Frequency	Proportion		
Pimeliinae	*Scytosoma* *pygmaeum*	SCP	6	8.11	4	1.79	5	4.57	5.03	2.14
	*Scytosoma dissilimarginis*	SCD	4	7.71	4	2.39	5	6.32	5.57	2.44
	*Microdera* *kraatzi*	MIK	6	50.30	5	32.26	6	23.52	36.66	2.53
	*Anatolica* *pandaroides*	ANP	5	2.74	5	17.20	5	24.33	13.71	2.28
	*Anatolica* *planata*	ANPL	2	3.14	6	18.40	5	30.65	16.09	2.22
	*Anatolica* *polita*	ANPO	2	3.65	4	7.65	6	6.85	5.88	2.85
Tenebrioninae	*Blaps* *femoralis*	BLF	6	4.87	6	15.05	4	0.81	7.01	1.78
	*Melanesthes rugipennis*	MER	0	0.00	2	0.96	0	0.00	0.31	1.00
	*Penthicus* *alashanicus*	PEA	6	1.22	6	1.91	2	0.27	1.17	2.23
	*Opatyum* *subaratum*	OPS	2	5.68	2	0.72	1	0.27	2.49	1.29
	*Scleropatrum horridum*	SCH	4	12.07	5	1.31	5	2.42	5.77	1.50
Diaperinae	*Crypticus rufipes*	CRR	2	0.51	1	0.36	0	0.00	0.31	1.88
Total			986		837		744		100.00	-

* Frequency refers to the occurrence frequency of a species during the six samplings from May to October.

## Data Availability

The raw data supporting the conclusions of this article will be made available by the authors upon request.

## References

[1] Ren G., Yu Y. (1999). Tenebrionidae Insects in the Deserts and Semi–Deserts of China.

[2] Zhang D., Chen X., He D. (2012). Species Diversity of Darkling Beetles in Desert Landscape and Their Value as Bioindicators. Appl. Entomol..

[3] Bartholomew A., Hafezi S.A., Karimi S. (2016). Effects of Habitat Complexity on the Abundance, Species Richness and Size of Darkling Beetles (Tenebrionidae) in Artificial Vegetation. Arid. Environ..

[4] Shen Z., Zhao J. (2007). Prediction of the Spatial Patterns of Species Richness Based on the Plant–Topography Relationship: An Application of GAMs Approach. Acta Ecol. Sin..

[5] Ma Y., Ma C., Ouyang F., Hang J., Shi Y. (2021). Response of Spatial Heterogeneity of Surface Beetles to Environmental Changes in the Loess Hilly Region. Northwest A F Univ. Nat. Sci. Ed..

[6] Wang M., Li X., Yang Y., Yang G. (2020). Communities Biodiversity of Ground–Dwelling Beetle and Its Relations with Environmental Factors in Helan Mountains, Northwestern China. Arid. Land. Resour. Environ..

[7] Yang G., Jia L., Zhang J., Yu Y. (2016). Distribution of Darkling Beetles and Its Relationships with Topography in Helan Mountain, Ningxia. Environ. Entomol..

[8] Liu J., Zhao W., Li F., Fang J. (2017). Effects of Microtopography Variation on the Distribution of Ground Darkling Beetles in a Sandy Desert Ecosystem. Arid Zone Res..

[9] Ford H., Evans B., Van Klink R., Skov M.W., Garbutt A. (2017). The Importance of Canopy Complexity in Shaping Seasonal Spider and Beetle Assemblages in Saltmarsh Habitats. Ecol. Entomol..

[10] Bartholomew A., El Moghrabi J. (2018). Seasonal Preference of Darkling Beetles (Tenebrionidae) for Shrub Vegetation Due to High Temperatures, Not Predation or Food Availability. Arid. Environ..

[11] Liu J., Li F., Liu C., Liu Q. (2012). Influences of Shrub Vegetation on Distribution and Diversity of a Ground Beetle Community in a Gobi Desert Ecosystem. Biodivers. Conserv..

[12] Allema B., Hemerik L., Rossing W.A.H., Groot J.C.J., van Lenteren J.C., van der Werf W. (2019). Dispersal of a Carabid Beetle in Farmland Is Driven by Habitat-Specific Motility and Preference at Habitat Interfaces. Entomol. Exp. Appl..

[13] Yang G., Wang Y., Wang M., Jia L. (2021). Niche and Interspecific Association of Darkling Beetles in a Desert Grassland of Alluvial Fans in Helan Mountain, Northwestern China. Acta Entomol. Sin..

[14] Padial A.A., Ceschin F., Declerck S.A.J., Meester L.D., Bonecker C.C., Lansac-Tôha F.A., Rodrigues L., Rodrigues L.C., Train S., Velho L.F.M. (2014). Dispersal Ability Determines the Role of Environmental, Spatial and Temporal Drivers of Metacommunity Structure. PLoS ONE.

[15] De Los Santos A., Gómez-González L.A., Alonso C., Arbelo C.D., De Nicolás J.P. (2000). Adaptive Trends of Darkling Beetles (Col. Tenebrionidae) on Environmental Gradients on the Island of Tenerife (Canary Islands). J. Arid. Environ..

[16] Krasnov B., Ward D., Shenbrot G. (1996). Body Size and Leg Length Variation in Several Species of Darkling Beetles (Coleoptera: Tenebrionidae) Along a Rainfall and Altitudinal Gradient in the Negev Desert (Israel). J. Arid. Environ..

[17] Hendrickx F., Maelfait J.-P., Desender K., Aviron S., Bailey D., Diekotter T., Lens L., Liira J., Schweiger O., Speelmans M. (2009). Pervasive Effects of Dispersal Limitation on Within- and Among-Community Species Richness in Agricultural Landscapes. Glob. Ecol. Biogeogr..

[18] Johnson A.R., Milne B.T., Wiens J.A. (1992). Diffusion in Fractcal Landscapes: Simulations and Experimental Studies of Tenebrionid Beetle Movements. Ecology.

[19] Mo D., Zhu Z., Wan L. (1999). The Alluvial Fans along the Eastern Foot of Helan Mountain. Acta Sci. Nat. Univ. Pekin..

[20] Shen A., Zhao N., Shi Y., Mi W., She J., Zhang F., Guo R., Wu T., Li Z., Li J. (2024). Soil Ecological Stoichiometry in Varied Micro-Topographies of an Alluvial Fan at Eastern Helan Mountains, Northwest China. J. Arid. Land..

[21] Chu G., Wang M., Zhang S. (2014). Spatial Point Patters of Anabasis Aphylla Populations in the Proluvial Fan of South Junggar Basin. Sci. Silvae Sin..

[22] Ding J., Zhang P., Zhang H., Li Z., Feng Y. (2020). Spatial Heterogeneity of Ephedra Przewalskii Populations in the Stony Desert in the Middle of Southern Foot of Tianshan Mountains, China. Chin. J. Appl. Ecol..

[23] Parker K.C. (1995). Effects of Complex Geomorphic History on Soil and Vegetation Patterns on Arid Alluvial Fans. J. Arid. Environ..

[24] Flores D.G., Suvires G.M., Ocaña R. (2017). Actividad Geomorfológica y Colonización Vegetal En Depósitos de Abanicos Aluviales Del Desierto Del Monte Central de Argentina. Cuad. Investig. Geográfica Geogr. Res. Lett..

[25] Yang X., Wang J., Xu M., Ali A., Xu Y., Lamb D., Duan L.-C., Yan K.-H., Yang S.-T. (2019). Effects of the Ephemeral Stream on Plant Species Diversity and Distribution in an Alluvial Fan of Arid Desert Region: An Application of a Low Altitude UAV. PLoS ONE.

[26] Fu S., Lu B., Ye Z. (2010). Effects of Rock Fragments on Runoff and Soil Erosion. J. Soil. Water Conserv..

[27] Pietrasiak N., Drenovsky R.E., Santiago L.S., Graham R.C. (2014). Biogeomorphology of a Mojave Desert Landscape—Configurations and Feedbacks of Abiotic and Biotic Land Surfaces during Landform Evolution. Geomorphology.

[28] Lv W., Qiu Y., Xie Z., Wang X., Wang Y. (2021). Effects of Mulch Gravels with Different Sizes on the Process of Water Infiltration. Res. Soil. Water Conserv..

[29] Hassanzadeh Bashtian M., Karimi A., Sepehr A., Lakzian A., Caballero E.R. (2024). Spatial Relationship of Landform Surface Features and Biocrusts, and Their Effect on Soil Microbial Biomass on an Alluvial Fan. Earth Surf. Process. Landf..

[30] Ríos-Casanova L., Valiente-Banuet A., Rico-Gray V. (2006). Ant Diversity and Its Relationship with Vegetation and Soil Factors in an Alluvial Fan of the Tehuacán Valley, Mexico. Acta Oecologica.

[31] Treonis A.M., Sutton K.A., Unangst S.K., Wren J.E., Dragan E.S., McQueen J.P. (2019). Soil Organic Matter Determines the Distribution and Abundance of Nematodes on Alluvial Fans in Death Valley, California. Ecosphere.

[32] Ren Y., Chen Y., Chen D., Zhang H. (2023). Spatial and Temporal Effects on the Value of Ecosystem Services in Arid and Semi-Arid Mountain Areas—A Case Study from Helan Mountain in Ningxia, China. Front. Ecol. Evol..

[33] Cheli G.H., Bosco T., Flores G.E. (2022). The Role of *Nyctelia circumundata* (Coleoptera: Tenebrionidae) on Litter Fragmentation Processes and Soil Fertility in Northeastern Arid Patagonia. Geoderma.

[34] Cheli G.H., Bosco T., Flores G.E. (2022). The Role of *Nyctelia dorsata* Fairmaire, 1905 (Coleoptera: Tenebrionidae) on Litter Fragmentation Processes and Soil Biogeochemical Cycles in Arid Patagonia. Ann. Zool..

[35] de Vega C., Arista M., Ortiz P.L., Herrera C.M., Talavera S. (2011). Endozoochory by Beetles: A Novel Seed Dispersal Mechanism. Ann. Bot..

[36] Chen L., Zhang F., Su J., Wen Y. (2023). Spatially Differentiated Characteristics of Vegetation Carbon Sequestration Function in Helan Mountain Area and Its Driving Factors. Acta Ecol. Sin..

[37] Ren G., Ba Y. (2010). Fauna of Soil Darkling Beetles in China Vol. 2 Tenty Riforms (Coleoptera: Tenebrionidae).

[38] Ren G., Yang X. (2006). Fauna of Soil Darkling Beetles in China Vol. 1 Opatriformes (Coleoptera: Tenebrionidae).

[39] Bao S. (2000). Soil Agrochemical Analysis.

[40] Qian G., Dong Z., Luo W., Feng Y., Wu B., Yang W. (2014). Gravel Morphometric Analysis Based on Digital Images of Different Gobi Surfaces in Northwestern China. J. Desert Res..

[41] Yang Y., Yang G., Wang J. (2017). Effects of Topographic Factors on the Distribution Pattern of Carabid Species Diversity in the Helan Mountains, Northwestern China. Acta Entomol. Sin..

[42] Ge F. (2008). Principle and Methods of Inesct Ecology.

[43] Begon M., Townsend C., Harper J. (2016). Ecology: From Individuals to Ecosystems.

[44] Baselga A. (2010). Partitioning the Turnover and Nestedness Components of Beta Diversity. Glob. Ecol. Biogeogr..

[45] Yang G., Yu Y., Wang X. (2011). Tendbrionidae Fauna and Ecological Distribution in Ningxia Helan Mountain Nature Reserve. J. Ningxia Univ. Nat. Sci. Ed..

[46] Maeno K., Nakamura S., Babah M. (2014). Nocturnal and Sheltering Behaviours of the Desert Darkling Beetle, *Pimelia senegalensis* (Coleoptera: Tenebrionidae), in the Sahara Desert. Afr. Entomol..

[47] Harrison S., Ross S., Lawton J. (1992). Beta Diversity on Geographic Gradients in Britain. J. Anim. Ecol..

[48] Tonkin J.D., Stoll S., Jähnig S.C., Haase P. (2016). Contrasting Metacommunity Structure and Beta Diversity in an Aquatic-Floodplain System. Oikos.

[49] Imeni S., Sadough H., Bahrami S., Mehrabian A., Nosrati K. (2021). Geomorphological Controls on Vegetation Changes: A Case Study of Alluvial Fans in Southwest of Miami City, Northeastern Iran. Arab. J. Geosci..

[50] Sepehr A., Hosseini A., Naseri K., Gholamhosseinian A. (2022). Biological Soil Crusts Impress Vegetation Patches and Fertile Islands over an Arid Pediment, Iran. J. Ecol. Environ..

[51] Soberón J.M. (2010). Niche and Area of Distribution Modeling: A Population Ecology Perspective. Ecography.

[52] Yuan P., Wang M., Wang Y., Yang G. (2022). Interspecific Association and Community Stability of Ground–Dwelling Beetle in a Desert Grassland of Alluvial Fan in Helan Mountain. Chin. J. Ecol..

[53] Fattorini S., Mantoni C., Di Biase L., Strona G., Pace L., Biondi M. (2020). Elevational Patterns of Generic Diversity in the Tenebrionid Beetles (Coleoptera Tenebrionidae) of Latium (Central Italy). Diversity.

[54] Parmenter R.R., Macmahon J.A. (1988). Factors Limiting Populations of Arid-Land Darkling Beetles (Coleoptera: Tenebrionidae): Predation by Rodents. Environ. Entomol..

[55] Ayal Y., Merkl O. (1994). Spatial and Temporal Distribution of Tenebrionid Species (Coleoptera) in the Negev Highlands, Israel. J. Arid. Environ..

